# Transformation and survival in patients with Waldenström macroglobulinemia: a population-based study

**DOI:** 10.1007/s12672-025-03946-6

**Published:** 2025-11-21

**Authors:** Yu Du, Xiaona Chang, Xiangxiang Li, Shugang Xing

**Affiliations:** 1 Department of Radiology, Tongji Hospital, Tongji Medical College, Huazhong University of Science and Technology, Wuhan, China; 2 Department of Pathology, Union Hospital, Tongji Medical College, Huazhong University of Science and Technology, Wuhan, China; 3 Department of Hematology, Tongji Hospital, Tongji Medical College, Huazhong University of Science and Technology, 1095 JieFang Avenue, Wuhan, 430030 China

**Keywords:** Waldenström macroglobulinemia, Transformation, Cumulative incidence, Risk factors, Survival outcome

## Abstract

**Background:**

Waldenström’s macroglobulinemia (WM) is a rare hematologic neoplasm characterized by an indolent clinical course. However, a significant proportion of patients undergo histological transformation to aggressive diffuse large B-cell lymphoma (DLBCL). Long-term population-based data on the transformation and survival are scarce. Based on the Surveillance, Epidemiology, and End Results (SEER) database, we performed this study to estimate the risk of transformation and outcomes of patients with WM.

**Methods:**

8191 patients with WM were retrieved from the SEER database. Competing risk methods were employed to evaluate the cumulative incidences and putative risk factors for transformation. Survival outcomes were analyzed using Kaplan-Meier and Cox proportional hazards regression.

**Results:**

The cumulative incidence rates for transformation at 5 and 10 years were 1.0% (95% CI, 0.8%–1.3%) and 1.9% (95% CI, 1.5%–2.2%), respectively. A substantial increase in total mortality was related to the time-dependent transformation (HR, 4.21 [95% CI, 3.36–5.26]; *P* < 0.001). Patients who presented with transformation > 24 months after WM diagnosis had a longer overall survival (OS) than those who experienced transformation within 24 months (5-year rate, 81.9% [95% CI, 74.7%–89.9%] *v* 42.7% [95% CI, 26.4%–69.2%]; *P* < 0.001). The OS was shorter for patients with DLBCL transformed from WM than for those with matched *de novo* DLBCL (5-year rate, 28.0% [95% CI, 20.5%–38.1%] *v* 49.5% [95% CI, 41.2%–59.4%]; *P* = 0.001).

**Conclusions:**

Histological transformation to DLBCL was associated with significantly increased mortality in patients with WM, and the survival outcomes substantially worse than matched *de novo* DLBCL cases.

**Supplementary Information:**

The online version contains supplementary material available at 10.1007/s12672-025-03946-6.

## Introduction

Lymphoplasmacytic lymphoma (LPL) represents an indolent yet incurable subtype of B-cell neoplasm, histopathologically distinguished by a heterogeneous proliferation of plasma cells, lymphoplasmacytoid cells, and small B lymphocytes. Waldenström macroglobulinemia (WM), a clinically significant subset of LPL, is defined by the concomitant presence of serum monoclonal immunoglobulin M (IgM) paraproteinemia and bone marrow infiltration by clonal lymphoplasmacytic cells, with approximately 95% of LPL cases meeting the diagnostic criteria for WM [1, 2]. Collectively, these entities constitute approximately 2% of all incident cases of non-Hodgkin lymphoma (NHL) at initial diagnosis, representing a relatively rare subset within the spectrum of lymphoid malignancies [3]. In light of the predominance of IgM-secreting LPL and given that WM represents the overwhelming majority of LPL cases, the nomenclature of WM will be adopted throughout this study to encompass both disease entities.

WM is generally characterized by an indolent clinical phenotype, typically associated with prolonged overall survival [4]. However, a subset of patients may experience an aggressive disease course. Histological transformation (HT) to higher-grade lymphoproliferative disorders, along with the development of secondary malignancies, have been established as independent prognostic determinants significantly correlated with adverse clinical outcomes in patients with WM [5–8]. Despite significant improvements in overall survival rates achieved through the sequential incorporation of rituximab-based immunotherapy and Bruton tyrosine kinase (BTK) inhibitors into the therapeutic armamentarium for WM, the clinical management of patients experiencing high-grade histological transformation continues to pose substantial therapeutic challenges and remains an area of unmet medical need [9, 10]. A significant limitation of existing literature [5, 6, 11, 12] investigating histological transformation in WM stems from the predominant reliance on small, single-institution patient cohorts, which may not be representative of the broader patient population. This methodological constraint has potentially led to incomplete characterization of the natural history of transformation, including its true incidence, associated clinical course, and long-term prognostic implications in the general WM population.

Diffuse large B-cell lymphoma (DLBCL) represents the most frequent histopathological variant observed in high-grade transformation events among patients with WM [7, 13]. Consequently, the present study will specifically focus on evaluating the risk of histological transformation to DLBCL and its prognostic impact on survival outcomes in patients with WM, utilizing comprehensive, population-level data derived from the Surveillance, Epidemiology, and End Results (SEER) program, a nationally representative cancer registry in the United States.

## Methods

### Patient selection

The SEER database gathers cancer data, including information on demographics and clinical features from population-based cancer registries. In this study, patients who were newly diagnosed with WM from January 2000 to December 2020, and were ≥ 18 years old were identified in the SEER database according to the *International Classification of Diseases for Oncology*,* Third Revision* (ICD-O-3) of the World Health Organization morphology codes 9671/3 and 9761/3. Cases without positive microscopic confirmation or positive laboratory test results, unknown survival data, or WM not identified as the first cancer were excluded from the study. Transformation from WM to DLBCL was diagnosed according to histologic confirmation, and cases demonstrating transformation within 2 months of WM diagnosis were not included in this study. DLBCL were identified using morphology code 9680/3. After the above-mentioned exclusions, 8191 patients with WM were found; 130 of them had transformation, and 3643 of them had died by the time the follow-up ended. For all patients, age at diagnosis, sex, race and ethnicity, Ann Arbor stage, months from diagnosis to treatment, treatment strategy, survival time and outcomes at the last follow-up, cause of death, year of diagnosis, and the interval from WM diagnosis to transformation were obtained. Patients were considered to have received deferred treatment if the interval between diagnosis and initial treatment was more than 3 months.

### Statistical analysis

Distributions of clinical and demographic characteristics were compared utilizing either Chi-squared or Fisher’s exact test according to transformation status.

Using competing risk methods [14], the cumulative incidences of transformation were calculated, with death from any reason regarded to be the sole competing risk. The period to the first event was measured from the moment of WM diagnosis until the occurrence of transformation or mortality. Patients who had no events at the last follow-up were censored. The cumulative incidence curves of transformation were compared using Gray’s tests [15]. To assess the impact of various factors on transformation, the sub-distribution hazard ratio (SHR) along with their corresponding 95% confidence interval (CI) were calculated by fitting the Fine and Gray’s proportional sub-distribution hazard models [16]. The period from WM diagnosis until death is termed overall survival 1 (OS_1_), while the interval from disease transformation to death is defined as overall survival 2 (OS_2_). Disease-specific survival (DSS) was defined as the period between the initial diagnosis of WM and death attributable to WM. The Kaplan–Meier method was used to depict the OS and DSS curves, which were subsequently compared utilizing the log-rank tests. The impact of putative predictors on OS and DSS was assessed using univariate and multivariate Cox proportional hazard regression models.

A comparative study using case-matched analysis was carried out to evaluate the survival outcomes of patients whose DLBCL originated from a transformation of WM against those diagnosed with *de novo* DLBCL. Cases with DLBCL as the first malignancy were identified from the SEER database using the morphology code 9680/3. A 1:1 matching ratio was employed for patients with *de novo* DLBCL and those with transformation, based on age (± 2 years), sex, race, stage, B symptom presence, primary site, and year of diagnosis (± 2 years). The case-matched process was entirely random, disregarding factors such as the cause of death and survival status.

All statistical tests were two-sided, and differences with *P* < 0.05 were considered to be significant. The statistical analyses were conducted using STATA Release 16.0 (Stata-Corp LLC, College Station, TX, USA) and R version 4.2.3 (R Foundation for Statistical Computing, Vienna, Austria).

## Results

### Characteristics of patients with WM

8191 patients diagnosed with LPL (3507 [42.8%])/WM (4684 [57.2%]) were included in this study (Table 1). At the time of diagnosis, the patients had a median age of 70 (range, 18–90 + years), with 63.5% being older than 65 years, and the male were a slight predominance (4830 [59.0%] male and 3361 [41.0%] female). Most of the patients were White (6796 [83.0%]). Excluding patients with an unknown stage, 86.1% of the patients were categorized as stage III/IV. A total of 312 (3.8%) patients had a deferred therapy. Chemotherapy was administered as a single modality in most patients (44.5%), followed by radiotherapy (1.4%), and a combined modality (0.8%). The remaining patients received no or unknown treatments (53.3%).

**Table 1:** Clinical characteristics of patients with WM

Variable and Category	Total		Non-transformation		Transformation		*P*
	No.	%	No.	%	No.	%	
Total No. of patients	8191	100.0	8061	98.4	130	1.6	
Histology							0.513
LPL	3507	42.8	3455	98.5	52	1.5	
WM	4684	57.2	4606	98.3	78	1.7	
Age at diagnosis, years							0.397
≤ 65	2986	36.5	2934	98.3	52	1.7	
> 65	5205	63.5	5127	98.5	78	1.5	
Sex							0.906
Male	4830	59.0	4754	98.4	76	1.6	
Female	3361	41.0	3307	98.4	54	1.6	
Race and Ethnicity							0.394
NHW	6796	83.0	6687	98.4	109	1.6	
NHB	373	4.6	368	98.7	5	1.3	
NHAIAN	22	0.3	22	100	0	0	
NHAPI	455	5.6	444	97.6	11	2.4	
Hispanic	545	6.7	540	99.1	5	0.9	
Ann Arbor stage							0.329
Ⅰ	239	2.9	237	99.2	2	0.8	
Ⅱ	86	1.0	84	97.7	2	2.3	
Ⅲ	120	1.5	118	98.3	2	1.7	
Ⅳ	1889	23.1	1850	97.9	39	2.1	
Unknown	5857	71.5	5772	98.5	85	1.5	
Deferred therapy							0.950
Yes	312	3.8	307	98.4	5	1.6	
No	4112	50.2	4045	98.4	67	1.6	
Unknown	3767	46.0	3709	98.5	58	1.5	
Treatment							0.524^b^
No treatment^a^	4369	53.3	4305	98.5	64	1.5	0.344^c^
CT only	3647	44.5	3584	98.3	63	1.7	
RT only	112	1.4	111	99.1	1	0.9	
CT + RT	63	0.8	61	96.8	2	3.2	
Year of diagnosis							0.113
2000–2010	3847	47.0	3777	98.2	70	1.8	
2011–2020	4344	53.0	4284	98.6	60	1.4	

Abbreviations: CT, Chemotherapy; LPL, Lymphoplasmacytic lymphoma; NHAIAN, Non-Hispanic American Indian/Alaska Native; NHAPI, Non-Hispanic Asian or Pacific Islander; NHB, Non-Hispanic Black; NHW, Non-Hispanic White; RT, Radiotherapy; WM, Waldenström macroglobulinemia

^a^The group with no treatment (no treatment, on active surveillance) included patients who had “unknown” chemotherapy or radiotherapy status. The Surveillance, Epidemiology and End Results database classifies chemotherapy (radiotherapy) data as “patients had chemotherapy (radiotherapy)” and “no/unknown”

^b^No treatment, CT only, RT only, CT + RT

^c^Any treatment vs. no treatment

### Transformation to DLBCL

**Fig. 1:**
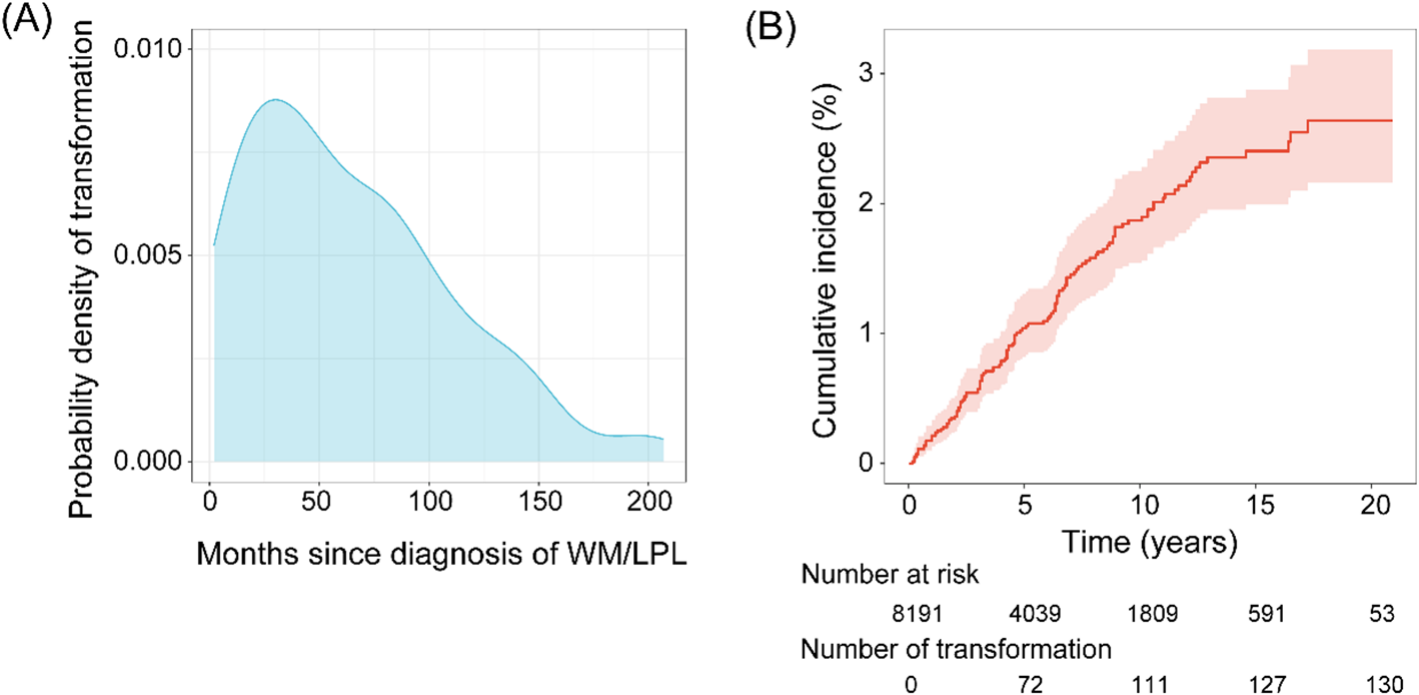
Transformation in patients with Waldenström macroglobulinemia. (A) Probability density curve of transformation; (B) Cumulative incidence of transformation for the entire cohort with corresponding 95% confidence intervals

Histologically confirmed transformation to DLBCL was diagnosed in 130 (1.6%) patients. Most transformation events occurred within 10 years of WM diagnosis (85.4%, Fig. 1A). The median duration from the diagnosis of WM to its transformation was 54.5 months (range, 2–207 months). With 3552 deaths considered as a competing risk and 130 transformation events occurring after WM diagnosis, the cumulative incidence rates for transformation were 1.0% (95% CI, 0.8%–1.3%) at 5 years and 1.9% (95% CI, 1.5%–2.2%) at 10 years (Fig. 1B). The corresponding estimated annual incidence rate for transformation was 2.6 per 1000 patient-years (95% CI, 2.2–3.1 per 1000 patient-years) (Table S1).

The results obtained from the univariate Fine and Gray’s proportional subdistribution hazards models for transformation were presented in Table 2. However, this investigation did not identify any statistically significant clinical or demographic factors demonstrating a substantial association with the risk of histological transformation in the studied cohort (Figure S1).

**Table 2:** Univariate analyses for risk factors of transformation, DSS, and OS_1_

Variable and category	Transformation		DSS		OS_1_	
	SHR (95% CI)	*P*	HR (95% CI)	*P*	HR (95% CI)	*P*
Transformation (time dependent)						
Yes *v* No	NA		NA		4.37 (3.49–5.46)	< 0.001
Age at diagnosis						
> 65 *v* ≤ 65 years	0.93 (0.65–1.31)	0.661	2.40 (2.15–2.67)	< 0.001	3.28 (3.03–3.55)	< 0.001
Sex						
Female *v* Male	1.01 (0.71–1.43)	0.959	0.83 (0.75–0.91)	< 0.001	0.84 (0.78–0.90)	< 0.001
Race and Ethnicity						
Hispanic	*Ref.*		*Ref.*		*Ref.*	
NHW	1.65 (0.67–4.04)	0.280	0.98 (0.81–1.20)	0.874	0.87 (0.76–0.99)	0.038
NHB	1.32 (0.38–4.55)	0.670	0.86 (0.64–1.16)	0.319	0.84 (0.69–1.03)	0.089
NHAIAN	NA	NA	1.74 (0.85–3.57)	0.131	0.96 (0.51–1.81)	0.906
NHAPI	2.61 (0.91–7.50)	0.075	0.98 (0.74–1.29)	0.876	0.87 (0.72–1.05)	0.136
Ann Arbor stage						
Ⅰ-Ⅱ	*Ref.*		*Ref.*		*Ref.*	
Ⅲ-Ⅳ	1.72 (0.61–4.81)	0.303	1.22 (0.96–1.55)	0.110	1.19 (1.02–1.39)	0.027
Missing	1.64 (0.60–4.49)	0.337	1.23 (0.97–1.55)	0.087	1.04 (0.89–1.20)	0.650
Deferred therapy						
Yes	*Ref.*		*Ref.*		*Ref.*	
No	1.01 (0.41–2.52)	0.976	1.41 (1.09–1.83)	0.009	1.30 (1.08–1.56)	0.005
Unknown	0.92 (0.37–2.31)	0.864	0.96 (0.74–1.25)	0.763	1.11 (0.93–1.33)	0.259
Treatment^a^						
Yes *v* No/Unknown	1.15 (0.82–1.63)	0.418	1.51 (1.37–1.66)	< 0.001	1.14 (1.07–1.22)	< 0.001

Abbreviations: CI, Confidence interval; DSS, Disease-specific survival; HR, Hazard ratio; NA, Not available; NHAIAN, Non-Hispanic American Indian/Alaska Native; NHAPI, Non-Hispanic Asian or Pacific Islander; NHB, Non-Hispanic Black; NHW, Non-Hispanic White; OS, Overall survival; SHR, Subdistribution hazard ratio; WM, Waldenström macroglobulinemia

^a^Patients who have undergone at least one treatment regimen are compared to those who have received no treatment or whose treatment status is unknown

### Survival and effect of transformation on OS

**Fig. 2:**
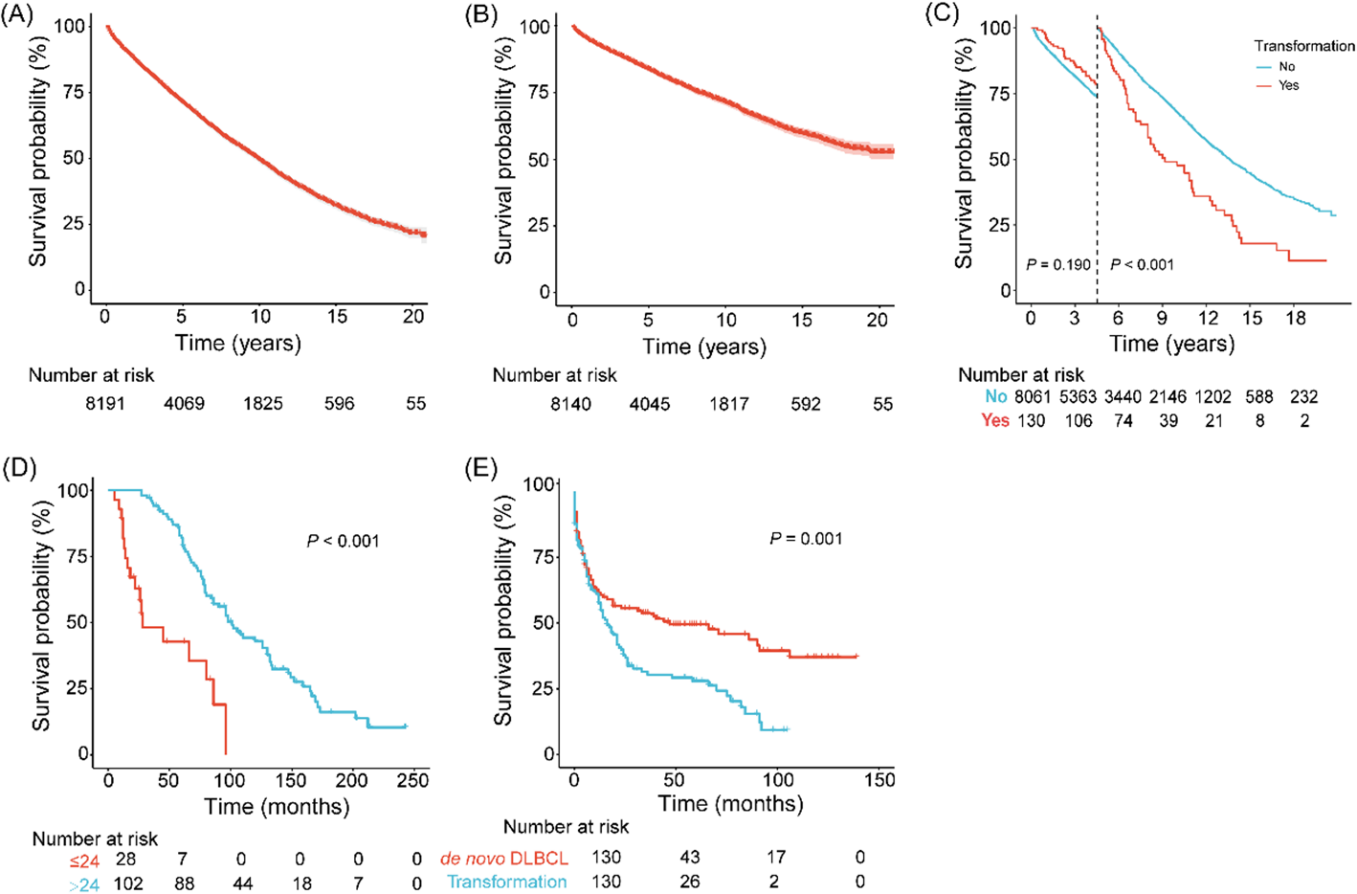
Kaplan–Meier curves for survival in patients with Waldenström macroglobulinemia. (A) OS_1_; (B) DSS; (C) OS_1_ for patients with and without transformation; (D) OS_1_ for patients with early and late transformation; (E) OS_2_ for patients with *de novo* DLBCL and those with DLBCL transformed from WM. DLBCL, diffuse large-B cell lymphoma; DSS, disease-specific survival; OS, overall survival

There were 3643 deaths during the 50,867 patient-years of follow-up (mortality rate, 71.6 per 1000 patient-years). The median OS_1_ was 119 months (95% CI, 115–123 months), whereas median DSS was not reached. The 5- and 10-year OS_1_ rates were 71.2% (95% CI, 70.2%–72.3%) and 49.5% (95% CI, 48.2%–50.9%), respectively (Fig. 2A). The 5- and 10-year DSS rates were 83.8% (95% CI, 82.9%–84.7%) and 71.4% (95% CI, 70.1%–72.8%), respectively (Fig. 2B).

Patients exhibiting transformation demonstrated a significantly shorter OS_1_ compared to those without transformation (10-year rate, 37.3% [95% CI, 29.2%–47.6%] *v* 49.8% [95% CI, 48.5%–51.2%], respectively; *P* < 0.001; Fig. 2C). Furthermore, patients whose transformation occurred more than 24 months after the initial diagnosis of WM exhibited a longer OS_1_ compared to those whose transformation took place within 24 months (5-year OS_1_ rates, 81.9% [95% CI, 74.7%–89.9%] *v* 42.7% [95% CI, 26.4%–69.2%], respectively; *P* < 0.001; Fig. 2D). The multivariate Cox proportional hazards regression indicated that time-dependent transformation was an independent predictor of decreased OS_1_ (HR, 4.21 [95% CI, 3.36–5.26], *P* < 0.001). As shown in Table 3, multivariate analyses incorporating significant individual variables also revealed that aged >65 years and males demonstrated statistically significant predictors of shorter DSS and OS_1_ (Figure S2 and S3). In this study, treatment might be associated with poorer DSS and OS_1_, most likely due to the fact that patients who underwent treatment initially presented with more aggressive disease and those without treatment were asymptomatic or minimally symptomatic [17].

**Table 3:** Multivariate analyses for risk factors of DSS and OS_1_ in patients with WM

Variable and category	DSS		OS_1_	
	HR (95% CI)	*P*	HR (95% CI)	*P*
Transformation (time dependent)				
Yes *v* No	NA		4.21 (3.36–5.26)	< 0.001
Age at diagnosis				
> 65 *v* ≤ 65 years	1.06 (1.05–1.06)	< 0.001	3.40 (3.14–3.68)	< 0.001
Sex				
Female *v* Male	0.78 (0.71–0.86)	< 0.001	0.80 (0.75–0.86)	< 0.001
Race and Ethnicity				
Hispanic				
NHW			0.82 (0.72–0.94)	0.003
NHB			1.06 (0.87–1.28)	0.592
NHAIAN			1.09 (0.58–2.05)	0.786
NHAPI			0.82 (0.68–0.99)	0.043
Ann Arbor stage				
Ⅰ-Ⅱ				
Ⅲ-Ⅳ			1.16 (0.99–1.36)	0.062
Missing			1.02 (0.88–1.19)	0.797
Deferred therapy				
Yes				
No	1.43 (1.10–1.85)	0.007	1.28 (1.06–1.53)	0.008
Unknown	1.25 (0.94–1.67)	0.126	1.11 (0.91–1.36)	0.311
Treatment				
Yes *v* No/Unknown	1.59 (1.37–1.86)	< 0.001	1.13 (1.01–1.25)	0.029

Abbreviations: CI, Confidence interval; DSS, Disease-specific survival; HR, Hazard ratio; NA, Not available; NHAIAN, Non-Hispanic American Indian/Alaska Native; NHAPI, Non-Hispanic Asian or Pacific Islander; NHB, Non-Hispanic Black; NHW, Non-Hispanic White; OS, Overall survival; WM, Waldenström macroglobulinemia

Characteristics of the 130 patients with DLBCL transformed from WM were summarized in Table 4. With a median age of 74 years (range, 48–90 + years), 21 of 77 (27.3%) patients exhibited B symptoms at transformation. 33 of 62 patients (53.2%) had stage Ⅲ to Ⅳ disease, and extranodal involvement was present in 41.5%. The most frequent site of extranodal involvement were bone marrow (9.2%), central nervous system (8.5%), gastrointestinal tract (6.2%), lungs (3.8%), and skin (3.8%).

**Table 4:** Characteristics of patients with DLBCL transformed from WM

Characteristics	*N*	%
Age (median, range), years	74 (48–90+)	
Sex (M/F)	76/54	58.5/41.5
Race		
White	114	87.7
Black	5	3.8
Other	11	8.5
Site		
Extranodal	54	41.5
Nodal	76	58.5
Stage		
Ⅰ	19	14.6
Ⅱ	10	7.7
Ⅲ	6	4.6
Ⅳ	27	20.8
Unknown	68	52.3
B symptom		
Yes	21	16.2
No	56	43.1
Unknown	53	40.8
Primary site		
Gastrointestinal tract	8	6.2
Lung	5	3.8
Skin	5	3.8
CNS	11	8.5
Bone marrow	12	9.2
Lymph node	76	58.5
Other	13	10.0
Survival		
Alive	39	30.0
Dead	91	70.0

Abbreviations: CNS, Central nervous system; DLBCL, Diffuse large B-cell lymphoma; F, Female; M, Male; WM, Waldenström macroglobulinemia

Among the 130 patients who underwent transformation, 39 survived and 91 died at the time of the last follow-up. The median OS_2_ was 16 months (95% CI, 12–23 months), and the OS_2_ rates were 37.8% (95% CI, 29.8%–47.9%) and 28.0% (95% CI, 20.5%–38.1%) at 2 years and 5 years, respectively (Fig. 2E). Comparative survival analysis revealed significantly inferior survival outcomes in patients with WM-transformed DLBCL when compared to matched cases of *de novo* DLBCL with comparable clinicopathological characteristics (5-year OS_2_ rates, 28.0% [95% CI, 20.5%–38.1%] *v* 49.5% [95% CI, 41.2%–59.4%], respectively; *P* = 0.001; Fig. 2E).

### Causes of death

**Fig. 3:**
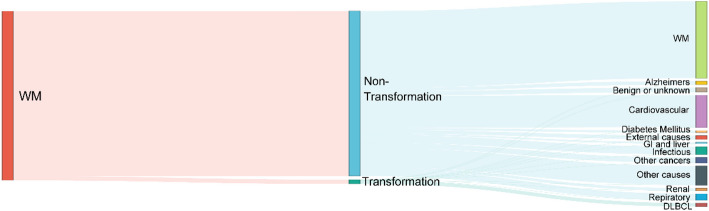
Sankey diagram shows the proportion of causes of death. DLBCL, diffuse large-B cell lymphoma; GI, Gastrointestinal; WM, Waldenström macroglobulinemia

During the study period, there were 3,643 deaths. The causes of death were illustrated in Fig. 3. Of these, 1,654 (45.4%) were attributed to WM, and 190 (5.2%) to secondary malignancies. Other leading causes of death included cardiovascular diseases (698, 19.2%), followed by infectious (166, 4.6%) and respiratory diseases (137, 3.8%). Of the 91 deaths among patients experiencing transformation, 74 (81.3%) were attributed to DLBCL.

**Discussion**.

As far as we know, this study based on population is the largest to investigate transformation in patients diagnosed with WM. Data from 8191 patients were examined, with a median follow-up of 107 months, during which 130 histologically confirmed transformations were diagnosed. The cumulative incidence rates for transformation were 1.0% at 5 years and 1.9% at 10 years. Compared with patients who did not experience transformation, transformation resulted in a significantly higher mortality rate. Additionally, we found that the OS was shorter for individuals with DLBCL transformed from WM than for those with *de novo* DLBCL.

In the present study, the cumulative incidence rates of transformation at 5-year and 10-year were 1.0% and 1.9%, respectively. A retrospective single-center study involving 1466 patients reported a cumulative incidence of transformation of 1.0% at 5 years and 2.4% at 10 years, similar to our results [5]. However, in another retrospective analysis by Zanwar et al. [18], the 5- and 10-year cumulative incidence rates of transformation was 2.4% and 4.7%, respectively, which were higher than those in our study. This observation may be attributed to the fact that the development of aggressive non-Hodgkin lymphoma (NHL) subtypes concurrent with WM was classified and analyzed as histological transformation events within the framework of this investigation.

The development of reliable predictive biomarkers for identifying patients with WM at elevated risk of histological transformation represents a critical unmet need in clinical practice; however, current evidence remains limited due to insufficient cohort sizes and lack of comprehensive molecular profiling in existing studies. In a study by Zanwar et al. [18], multivariate analysis showed that the sole independent predictor of transformation was MYD88^WT^ status. Another study also observed that patients with the MYD88^WT^ genotype were at an increased risk of transformation (HR, 19.8) [11]. According to Leleu et al. [19], purine analogs have been linked to a higher risk of transformation and emergence of myelodysplasia or acute myeloid leukemia. Conversely, no differences in transformation were found in a study comparing fludarabine and chlorambucil [20]. Consistent with our findings, the investigation conducted by Lin et al. [7] similarly failed to identify statistically significant clinical or molecular predictors associated with the risk of histological transformation in their study cohort, further highlighting the methodological challenges and knowledge gaps in this area of research. The current investigation was limited by insufficient documentation of clinicopathological characteristics in the WM cohort, which precluded comprehensive risk stratification and multivariate analysis of potential transformation predictors. These methodological constraints underscore the necessity for future large-scale, prospective studies incorporating detailed molecular profiling and standardized clinical data collection to elucidate the complex interplay of factors associated with histological transformation risk.

In accordance with previous reports, OS_1_ was worse for patients with WM who experience transformation than for those who did not. Castillo et al. [5] reported a much shorter median OS_1_ in patients with transformation than in those without transformation (8.7 vs. 16 years). With transformation as a time-dependent covariate, patients who experienced transformation exhibited a significantly higher risk (HR, 5.075) for death than those who did not experience transformation in a study by Zanwar et al. [18]. Additionally, we found that individuals with transformation occurring >24 months after WM diagnosis had higher OS rates than those with transformation occurring within 24 months. Similar findings were also reported in high-grade transformations of follicular lymphoma [21, 22] and marginal zone lymphoma [23, 24].

Using a case-control matching method, we observed that patients with *de novo* DLBCL had higher OS_2_ rates compared to those with DLBCL transformed from WM. Similar finding was reported by Castillo et al. [8]. Possible explanations for this finding include differences in disease biology and treatment regimens [25]. Another factor to consider is that most clinical trials designed for WM or *de novo* DLBCL do not include individuals with transformations.

In our study, we found that patients with DLBCL transformed from WM presented with a high rate of extranodal disease (41.5%), and the bone marrow (9.2%) was the main site, followed by central nervous system (CNS) (8.5%), gastrointestinal tract (6.2%), lungs (3.8%), and skin (3.8%). The finding was consistent with what Durot et al. reported [6]. Previous studies have demonstrated a correlation between the MYD88 L265P mutation and extranodal involvement in patients with *de novo* DLBCL [26–28]. And, a hallmark of WM is MYD88 L265P mutation [29], which may facilitate the localization of tumor cells to extranodal sites in transformed WM.

Advantages of the current study comprise its population-based design and use of SEER data spanning a 21-year period. However, we acknowledge that some limitations should be addressed. First, the study design precluded a central pathology review, which may have led to potential misclassifications. Second, the absence of detailed individual-level treatment data impeded our ability to elucidate the relationship between treatment and transformation. Finally, it is possible that in a small percentage of cases, the DLBCL component did not arise from a real histological transformation, but rather developed *de novo*.

In conclusion, the 10-year cumulative incidence rate for transformation to DLBCL in patients with WM was 1.9%. A significantly higher risk of death was linked to transformation to DLBCL, and the results appeared to be worse than those in the general population when DLBCL developed *de novo*. Despite comprehensive analysis, this study did not identify statistically significant risk factors associated with histological transformation. These findings underscore the critical need for future investigations incorporating advanced molecular profiling and larger, multicenter cohorts to establish reliable predictive biomarkers for transformation susceptibility, elucidate the underlying molecular mechanisms driving transformation, and develop targeted therapeutic strategies to improve clinical outcomes and survival rates in this high-risk patient population.

## Supplementary Information

Below is the link to the electronic supplementary material.

Supplementary Material 1

## Data Availability

The data underlying this article is publicly accessible from the Surveillance, Epidemiology, and End Results (SEER) database repository (https:/seer.cancer.gov) .

## References

[1] Vitolo U, Ferreri AJ, Montoto S. Lymphoplasmacytic lymphoma-Waldenstrom’s macroglobulinemia. Crit Rev Oncol Hematol. 2008;67:172–85. 18499469 10.1016/j.critrevonc.2008.03.008

[2] Askari E, Rodriguez S, Garcia-Sanz R. Waldenstrom’s macroglobulinemia: an exploration into the pathology and diagnosis of a complex B-cell malignancy. J Blood Med. 2021;12:795–807. 34512060 10.2147/JBM.S267938 PMC8416181

[3] Alaggio R, Amador C, Anagnostopoulos I, Attygalle AD, Araujo IBO, Berti E, et al. The 5th edition of the world health organization classification of haematolymphoid tumours: lymphoid neoplasms. Leukemia. 2022;36(7):1720–48. 35732829 10.1038/s41375-022-01620-2 PMC9214472

[4] Kyrtsonis MC, Vassilakopoulos TP, Angelopoulou MK, Siakantaris P, Kontopidou FN, Dimopoulou MN, et al. Waldenström’s macroglobulinemia: clinical course and prognostic factors in 60 patients. Experience from a single hematology unit. Ann Hematol. 2001;80:722–7. 11797112 10.1007/s00277-001-0385-8

[5] Castillo JJ, Gustine J, Meid K, Dubeau T, Hunter ZR, Treon SP. Histological transformation to diffuse large B-cell lymphoma in patients with Waldenstrom macroglobulinemia. Am J Hematol. 2016;91:1032–5. 27415417 10.1002/ajh.24477

[6] Durot E, Tomowiak C, Michallet AS, Dupuis J, Hivert B, Leprêtre S, et al. Transformed Waldenstrom macroglobulinaemia: clinical presentation and outcome a multi-institutional retrospective study of 77 cases from the French innovative leukemia organization (FILO). Br J Haematol. 2017;179:439–48. 28770576 10.1111/bjh.14881

[7] Lin P, Mansoor A, Bueso-Ramos C, Hao S, Lai R, Medeiros LJ. Diffuse large B-cell lymphoma occurring in patients with lymphoplasmacytic lymphoma/Waldenstrom macroglobulinemia clinicopathologic features of 12 cases. Am J Clin Pathol. 2003;120:246–53. 12931555 10.1309/R01V-XG46-MFCD-VNHL

[8] Castillo JJ, Olszewski AJ, Kanan S, Meid K, Hunter ZR, Treon SP. Survival outcomes of secondary cancers in patients with Waldenström macroglobulinemia: an analysis of the SEER database. Am J Hematol. 2015;90:696–701. 25963924 10.1002/ajh.24052

[9] Gertz MA, Waldenström macroglobulinemia. 2023 update on diagnosis, risk stratification, and management. Am J Hematol. 2023;98(2):348–58. 36588395 10.1002/ajh.26796 PMC10249724

[10] Durot E, Tomowiak C, Michallet A-S, Dupuis J, Lepretre S, Toussaint E et al. Retrospective analysis of 56 cases of transformed Waldenström macroglobulinemia. a study on behalf of the French Innovative Leukemia Organization (FILO). Blood. 2016;128:2982 (abstract).

[11] Treon SP, Gustine J, Xu L, Manning RJ, Tsakmaklis N, Demos M, et al. MYD88 wild-type Waldenstrom macroglobulinaemia: differential diagnosis, risk of histological transformation, and overall survival. Br J Haematol. 2018;180(3):374–80. 29181840 10.1111/bjh.15049

[12] Berendsen MR, van Bladel DAG, Hesius E, Berganza Irusquieta C, Rijntjes J, van Spriel AB, et al. Clonal relationship and mutation analysis in lymphoplasmacytic lymphoma/Waldenström macroglobulinemia associated with diffuse large B-cell lymphoma. Hemasphere. 2023;7(11):e976. 37928625 10.1097/HS9.0000000000000976 PMC10621888

[13] Owen RG, Bynoe AG, Varghese A, de Tute RM, Rawstron AC. Heterogeneity of histological transformation events in Waldenström’s macroglobulinemia (WM) and related disorders. Clin Lymphoma Myeloma Leuk. 2011;11(1):176–9. 21856554 10.3816/CLML.2011.n.042

[14] Pintilie M. Competing risks: A practical perspective. Chichester, UK, John Wiley & Sons,; 2006. 10.1002/9780470870709.

[15] Gray R. A class of K-sample tests for comparing the cumulative incidence of a competing risk. Ann Stat. 1988;16:1141–54.

[16] Fine JP, Gray RJ. A proportional hazards model for the subdistribution of a competing risk. J Am Stat Assoc. 1999;94:496–509.

[17] Kumar SK, Callander NS, Adekola K, Anderson LD Jr, Baljevic M, Baz R, et al. Waldenström macroglobulinemia/lymphoplasmacytic lymphoma, version 2.2024, NCCN clinical practice guidelines in oncology. J Natl Compr Canc Netw. 2024;22(1D):e240001. 38244272 10.6004/jnccn.2024.0001

[18] Zanwar S, Abeykoon JP, Durot E, King R, Perez Burbano GE, Kumar S, et al. Impact of MYD88L265P mutation status on histological transformation of Waldenström macroglobulinemia. Am J Hematol. 2020;95(3):274–81. 31814157 10.1002/ajh.25697

[19] Leleu X, Soumerai J, Roccaro A, Hatjiharissi E, Hunter ZR, Manning R, et al. Increased incidence of transformation and myelodysplasia/acute leukemia in patients with Waldenström macroglobulinemia treated with nucleoside analogs. J Clin Oncol. 2009;27(2):250–5. 19064987 10.1200/JCO.2007.15.1530

[20] Leblond V, Johnson S, Chevret S, Copplestone A, Rule S, Tournilhac O, et al. Results of a randomized trial of Chlorambucil versus fludarabine for patients with untreated Waldenström macroglobulinemia, marginal zone lymphoma, or lymphoplasmacytic lymphoma. J Clin Oncol. 2013;31(3):301–7. 23233721 10.1200/JCO.2012.44.7920

[21] Link BK, Maurer MJ, Nowakowski GS, Ansell SM, Macon WR, Syrbu SI, et al. Rates and outcomes of follicular lymphoma transformation in the immunochemotherapy era: a report from the university of Iowa/MayoClinic specialized program of research excellence molecular epidemiology resource. J Clin Oncol. 2013;31(26):3272–8. 23897955 10.1200/JCO.2012.48.3990 PMC3757293

[22] Federico M, Caballero Barrigón MD, Marcheselli L, Tarantino V, Manni M, Sarkozy C, et al. Rituximab and the risk of transformation of follicular lymphoma: a retrospective pooled analysis. Lancet Haematol. 2018;5(8):e359–67. 30078408 10.1016/S2352-3026(18)30090-5

[23] Alderuccio JP, Zhao W, Desai A, Gallastegui N, Ramdial J, Kimble E, et al. Risk factors for transformation to higher-grade lymphoma and its impact on survival in a large cohort of patients with marginal zone lymphoma from a single institution. J Clin Oncol. 2018;36:3370–80. 10.1200/JCO.18.00138 30312133

[24] Du Y, Wang Y, Li Q, Chang X, Shen K, Zhang H, et al. Transformation to diffuse large B-cell lymphoma and its impact on survival in patients with marginal zone lymphoma: A population-based study. Int J Cancer. 2024;154(6):969–78. 37874120 10.1002/ijc.34773

[25] Durot E, Kanagaratnam L, Zanwar S, Kastritis E, D’Sa S, Garcia-Sanz R, et al. A prognostic index predicting survival in transformed Waldenström macroglobulinemia. Haematologica. 2021;106(11):2940–6. 33179472 10.3324/haematol.2020.262899 PMC8561274

[26] Braggio E, Van Wier S, Ojha J, McPhail E, Asmann YW, Egan J, et al. Genome-Wide analysis uncovers novel recurrent alterations in primary central nervous system lymphomas. Clin Cancer Res. 2015;21(17):3986–94. 25991819 10.1158/1078-0432.CCR-14-2116 PMC4558226

[27] Kraan W, Horlings HM, van Keimpema M, Schilder-Tol EJ, Oud ME, Scheepstra C, et al. High prevalence of oncogenic MYD88 and CD79B mutations in diffuse large B-cell lymphomas presenting at immune-privileged sites. Blood Cancer J. 2013;3(9):e139. 24013661 10.1038/bcj.2013.28 PMC3789201

[28] Rovira J, Karube K, Valera A, Colomer D, Enjuanes A, Colomo L, et al. MYD88 L265P mutations, but no other variants, identify a subpopulation of DLBCL patients of activated B-cell origin, extranodal involvement, and poor outcome. Clin Cancer Res. 2016;22(11):2755–64. 26792260 10.1158/1078-0432.CCR-15-1525

[29] Landgren O, Tageja N. MYD88 and beyond: novel opportunities for diagnosis, prognosis and treatment in Waldenström’s macroglobulinemia. Leukemia. 2014;28(9):1799–803. 24573383 10.1038/leu.2014.88

