# Supplementary material for: Transformation and survival in patients with Waldenström macroglobulinemia: a population-based study
Source: Discov Oncol. 2025 Nov 21;16:2137. doi: 10.1007/s12672-025-03946-6 (PMC12638563; doi:10.1007/s12672-025-03946-6)
Supplement: Supplementary file 1 — Supplementary Material 1 [file 12672_2025_3946_MOESM1_ESM.docx]

**Transformation and survival in patients with Waldenström macroglobulinemia: a population-based study**

Yu Du^1^; Xiaona Chang^2^; Xiangxiang Li^2^, Shugang Xing^3^

**Table S1.** Characteristics of follow-up in patients with WM.

**Figure S1.** Cumulative incidences of transformation in 8191 patients with Waldenström macroglobulinemia. (A) age; (B) sex; (C) race and ethnicity; (D) Ann Arbor stage; (E) deferred treatment versus no deferral; (F) treatment versus not/unknown.

**Figure S2.** Kaplan–Meier curves for overall survival in 8191 patients with Waldenström macroglobulinemia. (A) age; (B) sex; (C) race and ethnicity; (D) Ann Arbor stage; (E) deferred treatment versus no deferral; (F) treatment versus not/unknown.

**Figure S3.** Kaplan–Meier curves for disease-specific survival in 8140 patients with Waldenström macroglobulinemia. (A) age; (B) sex; (C) race and ethnicity; (D) Ann Arbor stage; (E) deferred treatment versus no deferral; (F) treatment versus not/unknown. *At the end of follow-up, 51 patients with unknown causes of death were excluded.

Table S1. Characteristics of follow-up in patients with WM

| Characteristics | Total |
| --- | --- |
| No. of patients (%) | 8191 (100.0) |
| Median age at diagnosis (range) | 70 (18-90+) |
| Male/Female | 4830/3361 |
| Median year of diagnosis (range) | 2011 (2000-2020) |
| Median follow-up months (95% CI) | 107 (104-111) |
| Follow-up time, patient-years (overall survival) | 50867 |
| No. of death (%) | 3643 (44.5) |
| Mortality rate (per 1000 patient-years) | 71.6 |
| Follow-up time, person-years (transformation) | 50614 |
| No. of transformation (%) | 130 (1.6) |
| Transformation rate (per 1000 patient-years) | 2.6 |

Abbreviations: WM, Waldenström macroglobulinemia.


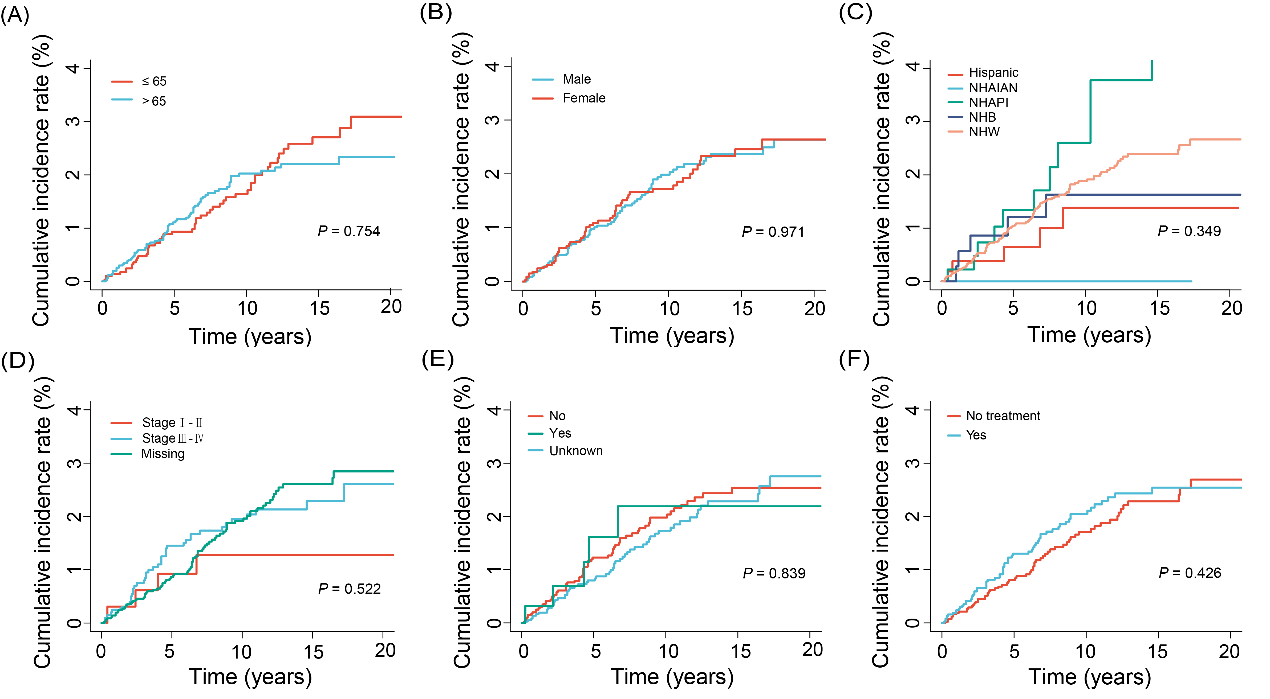


Figure S1. Cumulative incidences of transformation in 8191 patients with Waldenström macroglobulinemia. (A) age; (B) sex; (C) race and ethnicity; (D) Ann Arbor stage; (E) deferred treatment versus no deferral; (F) treatment versus not/unknown.


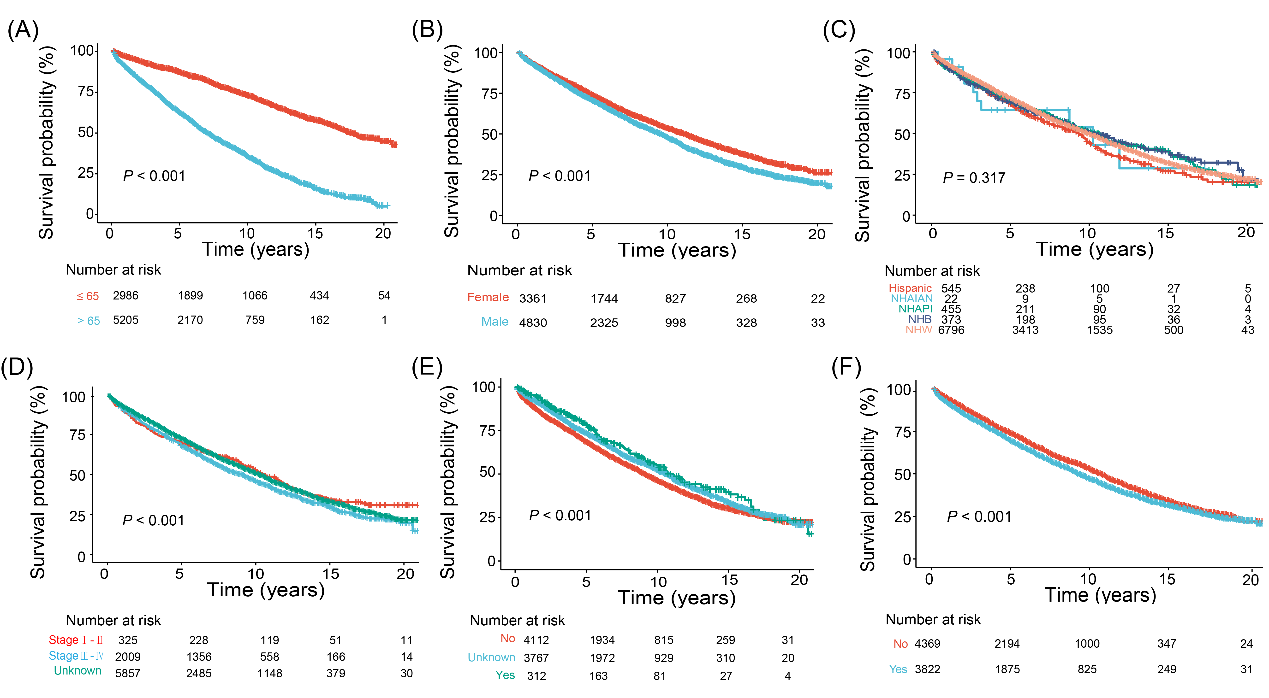


Figure S2. Kaplan–Meier curves for overall survival in 8191 patients with Waldenström macroglobulinemia. (A) age; (B) sex; (C) race and ethnicity; (D) Ann Arbor stage; (E) deferred treatment versus no deferral; (F) treatment versus not/unknown.


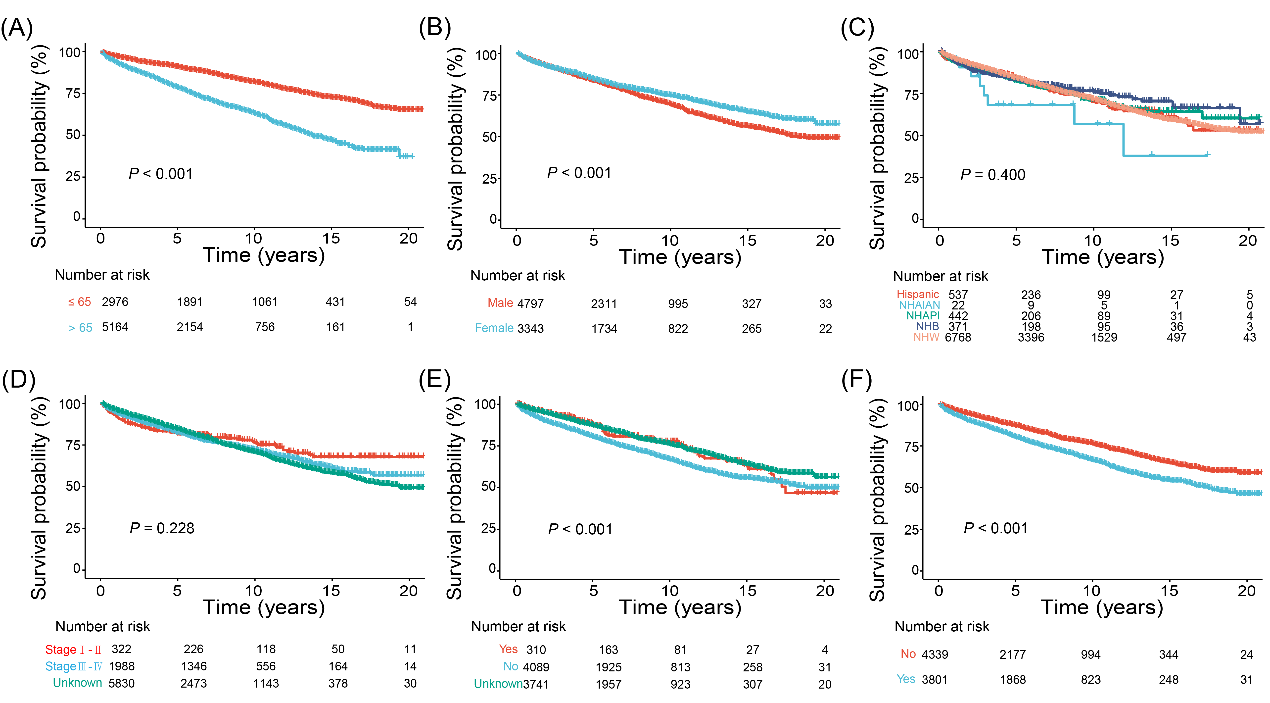


Figure S3. Kaplan–Meier curves for disease-specific survival in 8140 patients with Waldenström macroglobulinemia. (A) age; (B) sex; (C) race and ethnicity; (D) Ann Arbor stage; (E) deferred treatment versus no deferral; (F) treatment versus not/unknown. *At the end of follow-up, 51 patients with unknown causes of death were excluded.
